# Antimicrobial Selection for the Treatment of Clinical Mastitis and the Efficacy of Penicillin Treatment Protocols in Large Estonian Dairy Herds

**DOI:** 10.3390/antibiotics11010044

**Published:** 2021-12-30

**Authors:** Anri Timonen, Marju Sammul, Suvi Taponen, Tanel Kaart, Kerli Mõtus, Piret Kalmus

**Affiliations:** 1 Faculty of Veterinary Medicine, University of Helsinki, Yliopistonkatu 3, 00014 Helsinki, Finland; suvi.taponen@helsinki.fi; 2 Institute of Veterinary Medicine and Animal Science, Estonian University of Life Sciences, Kreutzwaldi 62, 51006 Tartu, Estonia; marju.sammul@ravimiamet.ee (M.S.); tanel.kaart@emu.ee (T.K.); kerli.motus@emu.ee (K.M.); piret.kalmus@emu.ee (P.K.); 3 State Agency of Medicines, Nooruse 1, 50411 Tartu, Estonia

**Keywords:** dairy cow, clinical mastitis, antimicrobial

## Abstract

Clinical mastitis (CM) is the most common microbial disease treated in dairy cows. We analyzed the antimicrobial usage in cows with CM (*n* = 11,420) in large dairy herds (*n* = 43) in Estonia. CM treatment data were collected during a 12-month study period. The antimicrobial usage was observed during the 21 days from the initiation of treatment, and the incidence of antimicrobial-treated CM was calculated for each study herd. The effect of intramammary (IMM), systemic, and combined (systemic and IMM) penicillin treatment of CM on the post-treatment somatic cell count (SCC) was analyzed using the treatment records of 2222 cows from 24 herds with a mixed multivariable linear regression model. The median incidence of antimicrobial-treated CM was 35.8 per 100 cow-years. Procaine benzylpenicillin and marbofloxacin were used in 6103 (35.5%, 95% CI 34.8–36.2) and 2839 (16.5%, 95% CI 16.0–17.1) CM treatments, respectively. Post-treatment SCC was higher after IMM penicillin therapy compared to systemic or combination therapy. Treatment of CM usually included first-choice antimicrobials, but different antimicrobial combinations were also widely used. The effect of procaine benzylpenicillin to post-treatment SCC was dependent on the administration route, cow parity, and days in milk. Further studies should evaluate the factors affecting veterinarians’ choice of antimicrobial used in the treatment of CM.

## 1. Introduction

Mastitis, an inflammation of the mammary gland caused by various bacterial species, is the primary reason for antimicrobial use on dairy farms [1,2]. International and national guidelines direct antimicrobial usage in veterinary medicine and instruct the treatment of clinical mastitis (CM) with antimicrobial products [3,4,5,6,7]. Mastitis treatment protocols of the antimicrobial compounds used, administration route, and treatment duration vary between geographical regions and countries [3,4,5,6,7]. The most common causative bacterial species of CM are *Staphylococcus aureus*, non-aureus staphylococci, streptococci, and coliforms [8,9]. A pathogen-specific approach using narrow-spectrum antimicrobials should be the most important goal of treatment [1,5,6], but their availability and the treatment guidelines and regulations in each country play a role in the treatment decisions in veterinary practice [10,11,12]. Several intramammary (IMM) and injectable antimicrobial products with different active ingredients are available in Estonia [13]. However, the national statistics of veterinary medicines of Estonia do not include information on their sales by animal species, and a centralized monitoring system for antimicrobial usage has not been implemented so far. While the use of critically important antimicrobials (CIA) is not prohibited in food-producing animals in Estonia, their use is strongly discouraged according to the national guidelines for antimicrobial treatment of production animals [14].

Revealing the efficacy of different mastitis treatment protocols is essential both for farmers and veterinarians. The resolution of CM can be assessed by a reduction in clinical signs, elimination of the causative pathogens, or a reduction in inflammation [15,16,17,18]. The milk somatic cell count (SCC) is commonly used in practice to evaluate the effectiveness of mastitis treatment as it will decrease gradually after bacteriological clearance; hence, a low post-treatment SCC level is the most important practical outcome of mastitis treatment [1]. Like the Nordic guidelines, the Estonian antimicrobial treatment guideline suggests procaine benzylpenicillin as the first-choice treatment of CM caused by Gram-positive penicillin-susceptible pathogens [5,6,13]. However, the antimicrobial compounds, dosages, and administration routes used for the treatment of CM in Estonian dairy cows are unknown. A 5-day treatment period of procaine benzylpenicillin administered systemically or intramammarily [19], or as a combination of these [20], has proven effective for the treatment of mastitis caused by Gram-positive penicillin-susceptible pathogens. In naturally occurring CM cases, 3–5 days of systemically administered benzylpenicillin treatment was efficient in young cows against *Staphylococcus aureus* mastitis, leading to lower milk SCC in bacteriologically cured quarters [21]. Similarly, treatment of chronic subclinical mastitis with systemic penethamate hydriodide, an ester of benzylpenicillin, resulted in lower SCC at both the cow and quarter levels 20 days post-treatment [22]. Yet, there is a lack of studies evaluating the effect of different penicillin administration routes on milk quality and udder health in field conditions of large, commercial dairy herds.

The aim of this study was to identify the annual incidence of antimicrobial-treated CM cases in cows of large, high-yielding Estonian dairy herds. The second purpose of this study was to analyze the usage of antimicrobials in treatment for CM in circumstances where different antimicrobial products are widely available for veterinarians. Additionally, we aimed to analyze the effect of different penicillin treatment protocols used for CM based on the post-treatment SCC.

## 2. Results

### 2.1. Incidence Rate of Antimicrobial-Treated CM and Number of Treatment Courses per Cow

In total, 11,420 antimicrobial treatment courses were implemented for 8554 dairy cows in 43 dairy herds during the 12-month study period. The median incidence rate of antimicrobial-treated CM across 43 farms was 35.8 (min 5.2 and max 150.2) cases per 100 cow-years.

Among the treated cows, 75.5% received one, 17.9% received two, and 6.6% received three CM treatment courses during the study period. In 63.3% of the treatment courses, only one treatment was used. Two treatments were used in 27.0% of the courses, and three or more were administered in 9.8% of the treatment courses.

### 2.2. Antimicrobial Compounds Used in the CM Treatments

In total, 17,180 antimicrobial treatments for CM were included in the study. The most used IMM products contained procaine benzylpenicillin, followed by ampicillin/cloxacillin and cefalexin/kanamycin. For the systemic administration, products containing procaine benzylpenicillin were used most, followed by products containing marbofloxacin or amoxicillin (Table 1).

### 2.3. Combination of Antimicrobials within a Treatment Course

**Figure 1:**
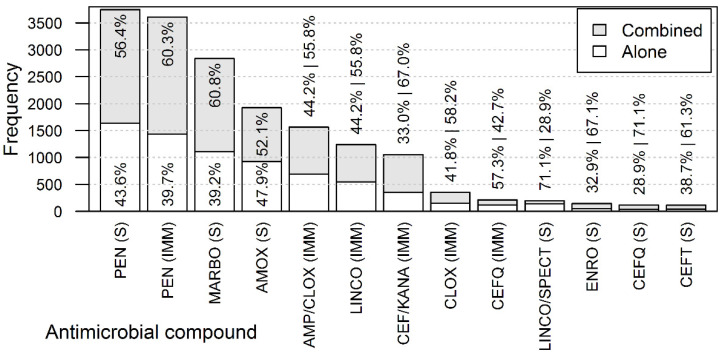
The number (height of bars) and percentage (numerical values) of antimicrobial compounds used as the only treatment per treatment course or used in combination with other compounds within the same treatment course. Systemic procaine benzylpenicillin (PEN_S); intramammary procaine benzylpenicillin (PEN_IMM); systemic marbofloxacin (MARBO_S); systemic amoxicillin (AMOX_S); intramammary ampicillin + cloxacillin (AMP/CLOX_IMM); intramammary lincomycin (LINCO_IMM); intramammary cefalexin + kanamycin (CEF/KANA_IMM); intramammary cloxacillin (CLOX_IMM); intramammary cefquinome (CEFQ_IMM); systemic lincomycin + spectinomycin (LINCO/SPECT_S); systemic enrofloxacin(ENRO_S); systemic cefquinome (CEFT_S CEFQ_S); systemic ceftiofur (CEFT_S).

The three most frequently used antimicrobial treatments—systemic procaine benzylpenicillin, IMM procaine benzylpenicillin, and systemic marbofloxacin—were solely used in approximately 40% of the treatment courses, while they were used in combination with other antimicrobial treatments in about 60% of the treatment courses (Figure 1).

During the first four days of the treatment course, systemic procaine benzylpenicillin was combined with IMM procaine benzylpenicillin in 44.9% of the cases and combined with systemic marbofloxacin in 14.1% of the cases. IMM cefalexin/kanamycin was mostly (37.3%) combined with systemic marbofloxacin (Table S1).

### 2.4. The Effect of Different Penicillin Treatment Schemes of CM on Post-Treatment Milk SCC

**Figure 2:**
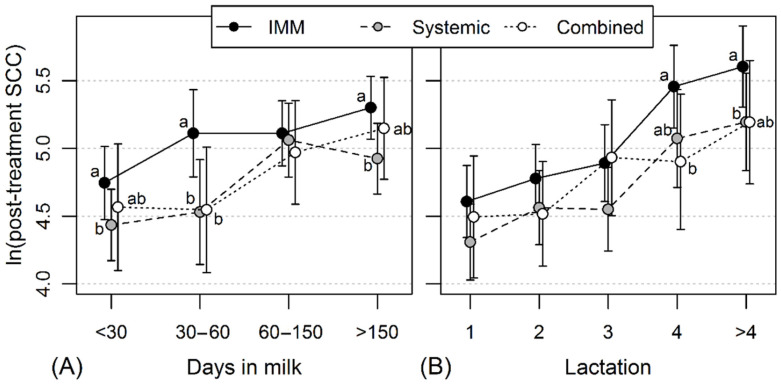
Model-based means of logarithm-transformed somatic cell count (SCC) at the closest post-treatment test milking depending on the penicillin treatment scheme (intramammary (IMM)/systemic/combined penicillin) and (**A**) lactation stage at treatment or (**B**) lactation number at treatment. Error lines denote the 95% confidence intervals and means without common letter at the same days in milk or lactation are statistically significantly different (*p* < 0.05).

Out of the recorded 2608 penicillin treatments, 1216 (46.6%) were local IMM treatments, 844 (32.4%) were systemic, and 548 (21.0%) were combined penicillin treatments. Treatment type (*p* = 0.025), lactation stage (*p* < 0.001), and lactation number (*p* < 0.001) all had a significant effect on the post-treatment milk SCC. Overall, the post-treatment SCC of cows treated with IMM penicillin was significantly higher compared to that of systemically treated cows (*p* = 0.007); a similar but nonsignificant (*p* = 0.287) difference was also observed between IMM and combined penicillin treatments. The post-treatment SCC at the early lactation stage (<30 DIM and 30–60 DIM) was significantly lower compared with that at later lactation stages (all *p* < 0.001). The post-treatment SCCs were significantly (*p* < 0.05) higher in cows treated with IMM compared to those that received systemic treatment at >30, 30–60, and >150 DIM; however, the difference between the treatment schemes was non-significant at 60–150 DIM (Figure 2A). The post-treatment SCC of the first three lactations was significantly lower compared with that measured during the 4th and later lactations (all *p* < 0.001). The post-treatment SCC was not significantly affected by the penicillin treatment scheme in younger cows (parity < 4), but it was significantly lower in 4th parity cows treated with a combined penicillin scheme and for >4th parity cows treated with systemically administered penicillin compared to cows in these lactations who received IMM treatment (*p* < 0.05) (Figure 2B).

## 3. Discussion

### 3.1. Incidence Rate of Antimicrobial-Treated CM

This study retrospectively analyzed the use of antimicrobial treatments administered under field conditions for CM occurring in large dairy herds in Estonia, where herd veterinarians have wide access to different antimicrobial products and there are no regulations on antimicrobial use in production animals. The median incidence of antimicrobial-treated CM was 35.8 cases per 100 cow-years, ranging between 5.2 and 150.2 per 100 cow-years across the study herds. There was a substantial difference in the incidence of CM in the study herds, even though most of the udder health indicators in the study herds were acceptable. A target of less than 25 CM cases per 100 cow-years is suggested [23]. In other studies, the mean and median incidence of CM has ranged between 0 and 55.6 cases per 100 cow-years [24,25,26,27,28], which is in line with our study. Our dataset only included CM cases in which antimicrobials were used for treatment and lacks information on cows treated only with supportive care or non-steroidal anti-inflammatory drugs or cows that were untreated. According to the authors’ knowledge, few CM cases are treated with non-steroidal anti-inflammatory drugs in Estonian dairy herds. Therefore, the true incidence of CM may be slightly higher in the herds of this study.

### 3.2. Antimicrobial Usage in the Treatment of CM Cases

Even though there is extensive access to different antimicrobial products in Estonia, the most used antimicrobial was procaine benzylpenicillin administered either systemically, locally as IMM infusion, or as a combination of these, which is in line with the national clinical guidelines of antimicrobial usage [14]. Additionally, the Nordic and Finnish guidelines of mastitis treatment suggest the use of procaine benzylpenicillin for CM caused by penicillin-susceptible pathogens and highlight the importance of milk bacteriology in the treatment decision [5,6]. However, marbofloxacin was the second most used antimicrobial in this study, which may indicate a high incidence of CM with severe clinical signs, possibly caused by Gram-negative bacteria. Fluoroquinolones and cephalosporins are the only antimicrobials to have proven beneficial effects on mastitis caused by *Escherichia coli*, but their use is only recommended in mastitis cases with severe clinical signs to prevent bacteremia and unlimited growth of bacteria [14,29].

Although 75% of the cows treated for CM received only one treatment course during the 12-month study period, the antimicrobial was often changed during the treatment. Several antimicrobials targeting different microbial groups, such as systemic procaine benzylpenicillin and marbofloxacin or systemic marbofloxacin and IMM cephalexin/kanamycin, were used within one treatment course. In Estonia, many of the veterinarians use on-farm culturing of CM bacteria, which may explain the relatively frequent change of antimicrobial during the first four days of treatment. However, the change in antimicrobials did not appear to follow the national guideline, and some CIAs used in human medicine, such as fluoroquinolones [30], were used frequently. After the end of this study, in July 2021, Estonia limited the use of fluoroquinolones and 3rd and 4th generation cephalosporins, and sensitivity testing prior to their use in cases where pathogens are resistant to other antimicrobials became mandatory [31]. Further education about CM treatment should be provided to Estonian production animal veterinarians to increase compliance with the national and international clinical guidelines. In addition, further studies analyzing veterinarians’ arguments and factors affecting their decision-making in CM treatment protocols in Estonian dairy farms should be conducted.

### 3.3. Association between Different CM Penicillin Treatment Protocols and the Level of Post-Treatment Milk SCC

Overall, a higher post-treatment composite milk SCC was identified in cows treated with IMM procaine benzylpenicillin compared to that of cows treated with systemic or combined procaine benzylpenicillin. In a study by Kalmus et al. (2014), post-treatment SCC was higher after 5-day IMM treatment of CM with procaine benzylpenicillin compared to systemic 5-day treatment with the same antimicrobial, which is comparable to the results of this study [19]. When systemic administration is used, the antimicrobial diffuses to all udder quarters, which may decrease the post-treatment composite milk SCC as possible subclinical infections from the other quarters may also be cured. Even though the cure rate for IMM treatment may be lower compared to that for systemic treatment, IMM treatment was the most popular treatment scheme. It is less invasive and higher concentrations of antimicrobial can be reached in the milk compartment [32] with lower total amounts of antimicrobial used [33].

The higher post-treatment SCC after treatment with IMM procaine benzylpenicillin compared to that following systemic or combination penicillin treatment was limited to the first 60 DIM. During this period, the cow reaches the peak milk yield, which may result in a negative energy balance and impair immunity [34,35]. Additionally, post-partum udder edema may negatively affect the spread of the antimicrobial into the tissue [36]. At the later lactation stage (>60 DIM) and in older cows, systemic penicillin treatment was also superior to IMM treatment. Mastitis in later lactation and in older cows may be chronic and cause higher SCC level compared to that in early lactation or younger cows [37,38]. Additionally, older cows may have multiple chronically infected udder quarters, leading to a higher composite milk SCC. Chronic mastitis, especially when caused by *Staphylococcus aureus*, is usually not treatable with antimicrobials [39], and antimicrobial treatment in these cases is not recommended.

### 3.4. Limitations of the Study

The farms enrolled in this study participated voluntarily and shared their data about antimicrobials used in CM treatment. We acknowledge that the criteria for diagnosing and treating CM might vary across veterinarians and farms, possibly inducing heterogeneity in the data. Still, field data were needed for meeting the aims of this study regarding revealing the selection of antimicrobials for the CM treatment. Although the number of the study farms was modest, approximately 30,000 dairy cows were reared in the recruited study herds, representing about 35% of the Estonian dairy cow population. This provides a plausible preliminary overview about the antimicrobial usage in the treatment of cow CM in Estonian dairy herds. However, the results of this study should be carefully extrapolated to dairy herds in other countries, with different herd sizes, management practices, and available antimicrobials, keeping in mind the voluntary basis of participation in this study.

In this study, we analyzed antimicrobial use for the treatment of CM without knowledge of the causative pathogen or the antimicrobial resistance. On-farm culture methods can be used in farm diagnostics to facilitate treatment decisions [16] and are used in Estonian dairy herds in mastitis diagnostics. However, our data were collected retrospectively, and the credibility of the bacteriology could have been reduced due to lack of knowledge about the methods, expertise, or management of the diagnostic procedure of on-farm culturing. Similarly, the post-treatment SCC after procaine benzylpenicillin treatment of CM was evaluated without bacteriological results. Hence, we cannot conclude any pathogen-specific regimens of antimicrobial usage or outcomes of penicillin treatment in this study. Additionally, we did not control the SCC before the treatments. Therefore, the history and course of the mastitis of the cows included in this study is unknown, which may affect the outcome of the procaine benzylpenicillin treatment. In daily field conditions, veterinarians have a great deal of information available for mastitic cows that is used to make decisions on which treatment scheme to apply for specific cows. Therefore, factors other than the age and lactation stage of the cows should be included in future studies to make sound recommendations for the use of different penicillin treatment schemes.

## 4. Materials and Methods

### 4.1. Collection of CM Treatment Data

Between 2018 and 2019, invitations to participate in this study were sent to 70 large (with ≥100 dairy cows) dairy farms in Estonia. In total, 43 (61.4%) dairy farms agreed to share their CM treatment data from the preceding 12 months. The herd sizes ranged between 100 and 2398 dairy cows, with an average herd size of 660 dairy cows. An automatic milking system was used on 9 farms with herd sizes ranging from 100 to 1318 dairy cows, and on 77% (*n* = 33) of the dairy farms, cows were milked in a milking parlor. Summary statistics of the annual production level and udder health parameters are given in Table 2.

CM was diagnosed by the farm personnel based on visible signs of inflammation in the udder or milk. If the milk had abnormal viscosity (watery, thicker than normal), color (yellow, blood-tinged), or consistency (flakes, clots), or udder edema or pain reaction presented, CM was diagnosed. The antimicrobial product and treatment protocol was set by the farm-employed veterinarian. All antimicrobial-treatment data were registered in the farms’ registers. The following CM antimicrobial-treatment data were collected from each farm: cow ID, treatment initiation date, product(s) name(s), active ingredient(s), administration route (systemic, intramammary, or combination), daily dosage (mL or number of intramammary tubes), and duration of treatment in days. The data did not include information about the causative udder pathogen or the number of infected udder quarters.

### 4.2. Analysis of the Efficacy of Different Penicillin Treatment Protocols on Post-Treatment Milk SCC

A separate dataset was created for the analysis of the effect of penicillin treatment protocols on cow composite milk post-treatment SCC. Mastitis cases were categorized based on the administration routes of procaine benzylpenicillin (systemically, intramammarily, or a combination of the two). For each CM case, the following data were collected from the Estonian Livestock Performance Recording Ltd. (ELPR, Tartu, Estonia): cow lactation number, days in milk (DIM) at the initiation of the treatment, and individual cow composite milk SCC from three consecutive milk recordings after the treatment initiation together with the date of the milk recording.

### 4.3. Definitions of a CM Treatment and a Treatment Course

All calculations were performed at the cow level because information regarding the infected udder quarters was not available. The following definitions were used to analyze the annual incidence of antimicrobial-treated CM cases and the use of different antimicrobials for CM treatment in the study herds:

(1) CM treatment course: The treatment course was initiated with the first administration of an antimicrobial product. In the 21-day period following the initiation of the treatment course, all antimicrobial treatments were considered part of the same treatment course. Any antimicrobials administered after that 21-day period were considered a new course of treatment.

(2) CM treatment: Each administration of a different antimicrobial during the same treatment course was considered a new treatment. To evaluate the association between the procaine benzylpenicillin treatment protocol and post-treatment SCC, each occurrence of CM was considered as a new case if there were at least 28 days between episodes of mastitis in the same cow (7 days for the maximum duration of the treatment as estimated in the incomplete available data + 21 days for recovery).

### 4.4. Data Editing

The initial CM antimicrobial-treatment dataset contained information on 17,261 treatments. Treatment cases (*n* = 81) in which the same antimicrobial product was repeatedly used on the same cow during the registered duration of treatment were removed. The final dataset contained information of 17,180 treatments.

The initial dataset used for analyzing the effect of different penicillin treatment protocols on post-treatment milk SCC included 3518 treatment records from 24 dairy herds. Of these, 25 records were excluded because of missing data (lactation number in 5 records and DIM in 20 records). Additionally, 212 records were excluded because the interval between the mastitis cases was <28 days (not considered a new mastitis case). To estimate the recovery from mastitis based on the test-day milk SCC, test-day SCC values obtained between 28 and 61 days post-treatment were used. As milk testing in farms occurs 11 times a year, and to ensure that each cow could have a SCC record available, the higher test-day cut-off was set at 61 days (365 days/11 times + 28 days). In total, 673 observations were excluded due to absence of post-treatment SCC measured 28 to 61 days after treatment. Finally, 2608 mastitis treatment records from 2222 cows in 24 herds were used for the statistical analysis.

### 4.5. Statistical Analysis

The incidence of antimicrobial-treated CM cases was calculated for each study herd using the number of antimicrobial-treated CM cases in the last 12 months as the numerator and the number of cow-years in the respective period as the denominator, and the incidence is expressed as the number of cases per 100 cow-years.

The number of treatment courses per cow and the number of different treatments per treatment course were also calculated. To decrease the diversity of treatments, different veterinary products (38 in total) composed of the same antimicrobial compound(s) were analyzed together; for example, all intramammary products containing ampicillin and cloxacillin were considered as the same treatment in the analysis. Among intramammary products, the following antimicrobial compound(s) were used: procaine benzylpenicillin, ampicillin/cloxacillin, cloxacillin, lincomycin, lincomycin/neomycin, cefacetrile/rifaximin, cefalexin/kanamycin, and cefquinome. Products available for systemic use included procaine benzylpenicillin, procaine benzylpenicillin/dihydrostreptomycin, amoxicillin, oxytetracycline, cefquinome, ceftiofur, enrofloxacin, marbofloxacin, lincomycin/spectinomycin, and sulfadiazine/trimethoprim.

The overall frequency of the use of these antimicrobials was calculated, and their use within the same treatment course was studied; the number of antimicrobials used as the only treatment per treatment course, as well as the number of antimicrobials used in combination with other antimicrobials within the same treatment course, were counted. Several antimicrobial products, which were used within the first four days of the same treatment course, were considered combined CM treatments (the number of days was chosen according to the average duration of a treatment in the dataset: 3.8 days), and the frequencies of all used combined antimicrobials were calculated.

To study the associations between the penicillin treatment scheme and the post-treatment SCC, a mixed linear model with logarithm-transformed post-treatment SCC as the dependent variable, with the treatment (intramammary/systemic/combined penicillin), lactation stage (early lactation dairy cows as <30 DIM, cows in high milk yield period as 30–60 DIM, mid-lactation dairy cows as 60–150 DIM, and cows in late lactation as >150 DIM), and lactation (1st, 2nd, 3rd, 4th, 5th, and later lactations) as fixed categorical factors, was fitted. In addition, all two-way interactions (treatment*lactation stage, treatment*lactation, and lactation stage*lactation) were included, and the random effects of farm and cow were considered to account for possible non-zero covariances between measurements obtained in the same farm or on the same cow. The degrees of freedom in hypothesis tests were calculated using the Kenward–Roger method, and the model-based means of logarithm-transformed SCC was used for group comparisons. The R 4.0.3 (R Foundation for Statistical Computing, Vienna, Austria) packages ‘lme4′, ‘car’, ‘emmeans’, and ‘multcomp’ were used. Statistical significance was assumed at *p* ≤ 0.05.

## 5. Conclusions

Even though the number of antimicrobial products for veterinary usage in Estonia is wide, procaine benzylpenicillin was the most used antimicrobial treatment of CM. However, combined use of antimicrobials deviating from evidence-based therapy were applied. This suggests an urgent need to raise the awareness of production animal veterinarians about antimicrobial usage and to analyze the purposes of veterinarians in treatment regimen decisions. In procaine benzylpenicillin treatment, the lactation stage and parity of the cow, as well as the administration route, should be considered to enhance the treatment outcome and recovery from mastitis. Further studies could include more cow-based historical and bacteriological data to control for their effect on mastitis recovery to make sound recommendations for the veterinarians in the CM treatment with first-choice antimicrobials.

## Figures and Tables

**Table 1:** Antimicrobial compounds used in the clinical mastitis treatments. Antimicrobials are listed in order by antimicrobial groups of the main active compound in the product.

Active Compound(s) in the Antimicrobial Product	Number of Treatments (%; 95% Confidence Intervals)		
	Systemic	IMM ^1^	Total
Penicillins			
Procaine benzylpenicillin	2498 (27.4; 26.5–28.3)	3605 (44.8; 43.7–45.9)	6103 (35.5; 34.8–36.2)
Procaine benzylpenicillin/dihydrostreptomycin	1249 (13.7; 13.0–14.4)	*	1249 (7.3; 6.9–7.7)
Amoxicillin	1926 (21.1; 20.3–22.0)	*	1926 (11.2; 10.7–11.7)
Ampicillin/cloxacillin	*	1562 (19.4; 18.5–20.3)	1562 (9.1; 8.7–9.5)
Cloxacillin	*	354 (4.4; 4.0–4.9)	354 (2.1; 1.9–2.3)
Cephalosporins			
Cefalexin/kanamycin	*	1054 (13.1; 12.3–13.8)	1054 (6.1; 5.8–6.5)
Cefacetrile/rifaximin	*	30 (0.4; 0.3–0.5)	30 (0.2; 0.1–0.2)
Ceftiofur	111 (1.2; 1.0–1.5)	*	111 (0.6; 0.5–0.8)
Cefquinome	114 (1.2; 1.0–1.5)	211 (2.6; 2.3–3.0)	325 (1.9; 1.7–2.1)
Fluoroquinolones			
Marbofloxacin	2839 (31.1; 30.2–32.1)	*	2839 (16.5; 16.0–17.1)
Enrofloxacin	143 (1.6; 1.3–1.8)	*	143 (0.8; 0.7–1.0)
Lincosamides			
Lincomycin	*	347 (4.3; 3.9–4.8)	347 (2.0; 1.8–2.2)
Lincomycin/neomycin	*	889 (11.0; 10.4–11.7)	889 (5.2; 4.8–5.5)
Lincomycin/spectinomycin	197 (2.1; 1.9–2.5)	*	197 (1.1; 1.0–1.3)
Other antimicrobials			
Tetracycline	42 (0.5; 0.3–0.6)	*	42 (0.2; 0.2–0.3)
Sulfadiazine/trimethoprim	9 (0.1; 0.0–0.2)	*	9 (0.1; 0.02–0.1)
Total	9128 (100.0)	8052 (100.0)	17,180 (100.0)

^1^ Intramammary. * Antimicrobial was not used with this administration route.

**Table 2:** Overview of the herd and udder health characteristics (*n* = 43).

Characteristic	Mean (Standard Error)	Median	Range (Min; Max)
Herd size (*n* of cows)	660 (472.3)	566	100; 2398
305-day milk yield (kg)	10,702 (175.1)	10,698	7915; 13,226
Herd SCC (× 1000/mL)	212 (5.4)	191	128; 537
IMI rate ^1^	25.0 (1.6)	23.2	14.5; 63.5
New IMI rate ^2^	6.8 (0.3)	6.8	5.2; 8.2
Chronic IMI rate ^3^	18.2 (1.9)	16.4	8.6; 46.3

^1^ Proportion (%) of cows with somatic cell count (SCC) over the threshold (150,000 cells/mL) indicating intramammary infection (IMI). ^2^ Proportion (%) of cows acquiring new IMI between two consecutive milk recordings calculated as SCC shift from <150,000 cells/mL to >150,000 cells/mL. ^3^ Proportion (%) of cows persistently infected, SCC >150,000 cells/mL in more than two consecutive milk recordings. Data were collected from the Estonian Livestock Performance Recording Ltd. (ELPR) database.

## Data Availability

The data are not publicly available due to privacy policy.

## Supplementary Materials

The following are available online at https://www.mdpi.com/article/10.3390/antibiotics11010044/s1, Table S1: Simultaneous use of different antimicrobials within the first four days of treatment cases. The calculations are based on the treatment courses, where multiple antimicrobials were administered. The diagonal shows the number of the primary treatment cases and rows outside the diagonal present the proportion of treatment cases, where another antimicrobial was combined with the diagonal antimicrobial within the first four days of treatment course. The antimicrobials are ordered according to their overall use frequency.
