# Supplementary material for: Antimicrobial Selection for the Treatment of Clinical Mastitis and the Efficacy of Penicillin Treatment Protocols in Large Estonian Dairy Herds
Source: Antibiotics (Basel). 2021 Dec 30;11(1):44. doi: 10.3390/antibiotics11010044 (PMC8772812; doi:10.3390/antibiotics11010044)
Supplement: Supplementary file 1 [file antibiotics-11-00044-s001.zip › antibiotics-1505011-supplementary.pdf]

Article

Antimicrobial Selection for the Treatment of Clinical Mastitis and the Efficacy of Penicillin Treatment Protocols in Large Estonian Dairy Herds

Anri Timonen [1,*], Marju Sammul [2,3], Suvi Taponen [1], Tanel Kaart [2], Kerli Mõtus [2] and Piret Kalmus [2]

[1] Faculty of Veterinary Medicine, University of Helsinki, Yliopistonkatu 3, 00014 Helsinki, Finland; suvi.taponen@helsinki.fi
[2] Institute of Veterinary Medicine and Animal Science, Estonian University of Life Sciences, Kreutzwaldi 62, 51006 Tartu, Estonia; marju.sammul@ravimiamet.ee (M.S.); tanel.kaart@emu.ee (T.K.); kerli.motus@emu.ee (K.M.); piret.kalmus@emu.ee (P.K.)
[3] State Agency of Medicines, Nooruse 1, 50411 Tartu, Estonia
* Correspondence: anri.timonen@helsinki.fi

Supplementary Materials

Table S1. Combined use of different antimicrobials within the first four days of treatment cases. The calculations based on the treatment courses, where multiple antimicrobials were administered. The diagonal shows the number of the primary treatment cases and rows outside diagonal present the proportion of treatment cases, where another antimicrobial was combined with diagonal antimicrobial within the first four days of treatment course. The antimicrobials are ordered according to their overall use frequency (see Table 1).

Antimicrobial	PEN (S)	PEN (IMM)	MARBO (S)	AMOX (S)	AMP/ CLOX (IMM)	LINCO (IMM)	CEF/ KANA (IMM)	CLOX (IMM)	CEFQ (IMM)	LINCO/ SPECT (S)	ENRO (S)	CEFQ (S)	CEFT (S)
PEN (S)	1823	44.9%	14.0%	5.5%	11.3%	4.2%	1.8%	0.3%	0.3%	0.2%	0.7%	0.1%	0.1%
PEN (IMM)	45.7%	1792	16.0%	4.5%	6.4%	1.7%	6.3%	0.1%	0.1%	0.0%	0.1%	0.1%	0.3%
MARBO (S)	14.1%	15.8%	1808	7.4%	4.0%	4.8%	10.2%	0.2%	0.2%	0.0%	1.8%	0.1%	0.2%
AMOX (S)	13.0%	10.4%	17.4%	770	8.3%	8.1%	0.8%	1.6%	0.8%	0.9%	1.3%	0.3%	0.6%
AMP/CLOX (IMM)	27.5%	15.2%	9.6%	8.5%	750	2.4%	3.2%	2.3%	2.7%	0.0%	0.3%	3.5%	0.0%
LINCO (IMM)	19.0%	7.4%	21.2%	15.3%	4.4%	406	2.0%	0.5%	0.0%	0.0%	0.5%	1.0%	1.5%
CEF/KANA (IMM)	6.5%	22.6%	37.3%	1.2%	4.8%	1.6%	496	0.0%	0.2%	0.0%	0.2%	0.6%	0.4%
CLOX (IMM)	6.0%	2.4%	3.6%	14.3%	20.2%	2.4%	0.0%	84	0.0%	0.0%	0.0%	1.2%	0.0%
CEFQ (IMM)	8.3%	2.8%	5.6%	8.3%	27.8%	0.0%	1.4%	0.0%	72	1.4%	2.8%	23.6%	1.4%
LINCO/SPECT (S)	11.1%	0.0%	0.0%	25.9%	0.0%	0.0%	0.0%	0.0%	3.7%	27	0.0%	0.0%	0.0%
ENRO (S)	15.9%	2.4%	39.0%	12.2%	2.4%	2.4%	1.2%	0.0%	2.4%	0.0%	82	0.0%	0.0%
CEFQ (S)	2.0%	4.1%	4.1%	4.1%	53.1%	8.2%	6.1%	2.0%	34.7%	0.0%	0.0%	49	0.0%
CEFT (S)	5.1%	12.8%	7.7%	12.8%	0.0%	15.4%	5.1%	0.0%	2.6%	0.0%	0.0%	0.0%	39

Systemic procainbenzyl penicillin (PEN_S); intramammary procainbenzyl penicillin (PEN_IMM); systemic marbofloxacin (MARBO_S); systemic amoxicillin (AMOX_S); intramammary ampicillin+cloxacillin (AMP/CLOX_IMM); intramammary lincomycin (LINCO_IMM); intramammary celfalexin+ kanamycin (CEF/KANA_IMM); intramammary cloxacillin(CLOX_IMM); intramammary cefquinome (CEFQ_IMM); systemic lincomycin+ spectinomycin (LINCO/SPECT_S); systemic enrofloxacin(ENRO_S); systemic cefquinome (CEFT_S CEFQ_S); systemic ceftiofur (CEFT_S)
